# Supplementary material for: The evolutionary drivers and correlates of viral host jumps
Source: Nat Ecol Evol. 2024 Mar 25;8(5):960–71. doi: 10.1038/s41559-024-02353-4 (PMC11090819; doi:10.1038/s41559-024-02353-4)
Supplement: Supplementary file 1 — Supplementary Note, Methods and Figs. 1–6. [file 41559_2024_2353_MOESM1_ESM.pdf]

nature ecology & evolution

Article https://doi.org/10.1038/s41559-024-02353-4

The evolutionary drivers and correlates of viral host jumps

In the format provided by the authors and unedited

Contents

Supplementary Notes **2**

Higher frequency of anthroponotic than zoonotic jumps is robust to sampling biases........ 2

Supplementary Methods **4**

Host jump inference 4

Mutational distance and dN/dS calculation 5

Supplementary Figures **7**

Supplementary Figure 1. Effect of sampling biases on inferred anthroponotic-zoonotic ratio. 7

Supplementary Figure 2. Selection of Mash distance threshold. 8

Supplementary Figure 3. Assessing the quality of clique-level genome alignments. 9

Supplementary Figure 4. Illustration of host jump inference. 10

Supplementary Figure 5. Permutation test illustration. 11

Supplementary Figure 6. Linear regression model residuals. 12

References **12**

Supplementary Notes

Higher frequency of anthroponotic than zoonotic jumps is robust to sampling biases

Our estimates of the frequency of anthroponotic and zoonotic jumps across vertebrate-associated viral families rely on ancestral state reconstruction, which we note may be confounded by sampling biases. Given the high proportion of human sequences in our dataset (**Fig. 1a**), a main concern is that the likelihood of an ancestral node being reconstructed as human may be high, leading to an overestimation of the anthroponotic frequency. To assess the extent of this bias, we investigated how the estimated ratio of anthroponotic to zoonotic jumps is affected by the proportion of human-associated genomes in 'Coronaviridae_12', the viral clique comprising SARS-CoV-2 sequences. A high frequency of anthroponotic jumps, especially to farmed minks (*Neovison vison*)[1–3] and wild white-tailed deer (*Odocoileus virginianus*)[4–6] have been described. In fact, natural and experimental infections of SARS-CoV-2 have been documented for species from nearly every mammalian order. Meanwhile, only sporadic cases of animal-to-human transmission have been described, such as from pet hamsters in Hong Kong[7], farmed mink[2], white-tailed deer[8], and a captive lion[9]. The extensive surveillance of SARS-CoV-2 in humans and animals therefore presents the current best case study to determine the effects of sampling biases on our results, with the expectation that humans transmit SARS-CoV-2 more frequently to animals than they do to us.

In our dataset, the proportion of human-associated sequences within Coronaviridae_12 is 63% (1,000/1,579). We randomly subsampled the number of human genomes, yielding proportions between 1.7% and the observed value of 63.3%, and performed ancestral reconstruction to determine the ratio of anthroponotic to zoonotic jumps. We found that as the proportion of human-associated genomes increases, the ratio of anthroponotic to zoonotic jump also increases (**Supplementary Fig. 1a**). Notably, however, the anthroponotic-zoonotic ratio is always greater than one (i.e., the expectation for SARS-CoV-2) when the proportion of human genomes is higher than 7.9% (**Supplementary Fig. 1a**), indicating that inference of whether humans are a greater source or sink for SARS-CoV-2 is robust even to extreme sampling biases. Extrapolating this finding to the full viral dataset, we re-calculated the overall anthroponotic-zoonotic ratio after removing viral cliques that comprise less than 15% animal-associated genomes, but still found a higher frequency of anthroponotic versus zoonotic jumps (**Supplementary Fig. 1b**). Further, there is good evidence in the literature for anthroponotic transmission of the viral species associated to 13/17 of these viral cliques (**Supplementary Table 8**). Overall, these results indicate that our finding that there are more anthroponotic than zoonotic jumps across vertebrate viruses is robust to sampling biases.

Supplementary Table 8: Evidence from the literature reporting natural infection of animal hosts with viral species associated to cliques with less than 15% animal-associated genomes. Reference numbers are based on those listed in this Supplementary Information file.

Viral clique	Prop. animal genomes	Viral species	Recipient	Study
Orthomyxoviridae_4	0.002	Influenza B virus	*Sus scrofa*	*Ran et al.*[10]
			Rhizomys pruinosus	*He et al.*[11]
Pneumoviridae_6	0.01	Orthopneumovirus hominis	*Pangolins*	Ye et al.[12]
Pneumoviridae_4	0.01	Orthopneumovirus hominis	*Pangolins*	Ye et al.[12]
Orthomyxoviridae_11	0.02	Gammainfluenzavirus influenzae	*Sus scrofa*	Kimura et al.[13]
Adenoviridae_8	0.02	Human mastadenovirus A	*Pan troglodytes*	Zhou et al.[14]
Picornaviridae_25	0.02	Enterovirus A	*Mus musculus*	Experimental infection likely
Picornaviridae_24	0.02	Enterovirus B	*Rhinopithecus roxellana*	Tan et al.[15]
Paramyxoviridae_4	0.03	Respirovirus pneumoniae	*Pan troglodytes*<br>*Papio ursinus*<br>*Cercopithecus mitis*	Negrey et al.[16]
Pneumoviridae_2	0.03	Metapneumovirus hominis	*Gorrila beringei*	Mazet et al.[17]
			Pan troglodytes	Negrey et al.[16]
Adenoviridae_5	0.06	Human mastadenovirus C	*Gorilla gorilla*	Medkour et al.[18]
Caliciviridae_36	0.07	Norovirus	*Macaca mulatta*	Jiang et al.[19]
Sedoreoviridae_2	0.08	Rotavirus A	*Bos taurus*	Bwogi et al.[20]
			Sus scrofa	Bwogi et al.[20]
Picornaviridae_30	0.09	Salivirus A	*Pan*	Negrey et al.[21]
Sedoreoviridae_1	0.12	Rotavirus A	*Bos taurus*	Bwogi et al.[20]
			Sus scrofa	Bwogi et al.[20]
Togaviridae_1	0.13	Chikungunya virus	*Macaca fascicularis*	Sam et al.[22]
Herpesviridae_17	0.14	Human alphaherpesvirus	*Chlorocebus sabaeus*	No evidence
Arenaviridae_31	0.14	Mammarenavirus lassaense	*Mastomys natalensis*	No evidence
			Lophuromys sikapusi	No evidence

Supplementary Methods

Host jump inference

To illustrate the algorithm used for inferring putative host jumps and non-host jump lineages, we will use the clique 'Parvoviridae_58' that comprises mammalian bocapoviruses as an example (**Supplementary Fig. 4**). In this clique, seven host jump lineages were identified comprising four distinct host jump events (*Sus scrofa*→*Rattus norvegicus*; *R. norvegicus*→*Mustela lutreola*; *R. norvegicus*→*R. rattus*; *R. flavipectus*→*R. norvegicus*). The algorithm used to identify host jumps traverses from tip to root, identifying the first ancestral node that differs from the host state of the tip, based on the relative likelihood of host states. For example, the algorithm traverses from the tip KY927868.1 (*R. norvegicus*) to Node 4 (*R. norvegicus*) since the likelihood of the nodes encountered so far being *R. norvegicus* is two-fold higher than alternative host states. The algorithm then traverses to Node 3 for which no host state is two-fold more likely than alternative host states, indicating that the ancestral host state for this node is ambiguous, and the traverse continues. The subsequent node is Node 2, whose host state of *S. scrofa* is two-fold more likely than alternative host states. Here, the traverse terminates, and the host jump lineage identified spans the branches between Node 2 (*S. scrofa*) and KY927868.1 (*R. norvegicus*; **Supplementary Table 9**).

For non-host jump lineages, the algorithm traverses from tip to node until a host state transition is encountered, which is defined as the ancestral host state being different from the tip state, and the ancestral host state having a two-fold greater likelihood than alternative states. Tips that were initially inferred to be part of a host jump lineage, or whose only ancestor is the root node, are excluded. However, by definition, any ancestral node of the same host state as the tip of interest can be chosen to represent a non-host jump lineage. For example, the branches between KY489986.1 and Node 1 or between KY489986.1 and Node 2 can be chosen, but to minimise pseudo-replication, we randomly select one ancestral node for this tip, which happens to be Node 2 in this case. The lineage leading to OM274032.1 is discarded as the next ancestral node is the root node. The full list of host jump and non-host jump lineages identified via this algorithm are shown in **Supplementary Table 9**. We then calculate the number of host jumps as the number of distinct nodes where a host transition occurred for each host pair. In this case, there are seven host jump lineages comprising four distinct host jump events.

Mutational distance and dN/dS calculation

For each clique, we calculated the minimum tip-to-node distance for a host-jump and for a non-host jump lineage. The minimum mutational distance associated with a host jump and non-host jump in this case is 0.00605 and 0.000824 substitutions per site, respectively (**Supplementary Table 9**). For dN/dS, we removed all lineages where the genome-wide dN or dS value is zero. We then calculated the minimum dN/dS as a measure of the minimum extent of adaptation observed for this clique, which in this case is 0.0359 for host jump lineages. All non-host jump lineages identified here had either zero dN or dS values and so no minimum dN/dS value could be calculated for this clique. As a result, this clique was omitted from our dN/dS analyses as no non-host jump can be used as a suitable control.

Supplementary Table 9: Host jump and non-host jump lineages identified for the viral clique Parvoviridae_58. Minimum mutational distance and dN/dS values for this clique are underlined.

Ancestral node	Tip	Ancestral state	Observed state	Is host jump?	Mutational distance (subst./site)	dN	dS	dN/dS
Node2	KY927867.1	*S. scrofa*	*R. norvegicus*	Yes	0.204	0.0433	0.497	0.0872
Node2	KY927868.1	*S. scrofa*	*R. norvegicus*	Yes	0.204	0.0433	0.497	0.0872
Node2	KY927869.1	*S. scrofa*	*R. norvegicus*	Yes	0.200	0.0428	0.494	0.0867
Node2	KY927872.1	*S. scrofa*	*R. norvegicus*	Yes	0.193	0.0435	0.470	0.0927
Node6	KY927871.1	*R. norvegicus*	*R. rattus*	Yes	0.00605	0.000510	0.0142	0.0359
Node4	MF085373.1	*R. norvegicus*	*M. lutreola*	Yes	0.00938	0.00292	0.0276	0.106
Node8	KY927870.1	*R. norvegicus*	*R. flavipectus*	Yes	0.0126	0.00409	0.0444	0.0921
Node1	KY489985.1	*S. scrofa*	*S. scrofa*	No	0.000824	0	0.00206	N.A.
Node2	KY489986.1	*S. scrofa*	*S. scrofa*	No	0.00437	0	0	N.A.

Supplementary Figures

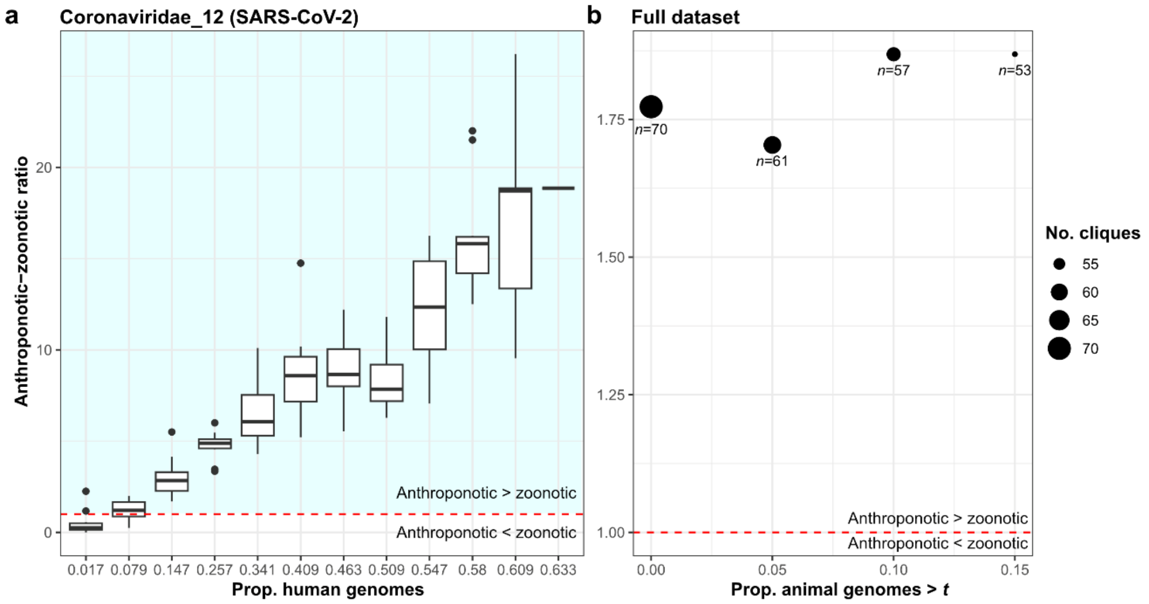

Supplementary Figure 1. Effect of sampling biases on inferred anthroponotic-zoonotic ratio. (a) Estimates of anthroponotic-zoonotic ratio following subsampling on Coronaviridae_12, the viral clique comprising SARS-CoV-2. Human-associated genomes were randomly subsampled to various degrees prior to ancestral state reconstruction. A total of 10 iterations of the analysis per human-genome proportion were performed. Boxplot elements are defined as follows: centre line, median; box limits, upper and lower quartiles; whiskers, 1.5x interquartile range. (b) Ratio of anthroponotic to zoonotic jumps when retaining only viral cliques with greater than a certain proportion of animal-associated genomes, *t*, in the full dataset.

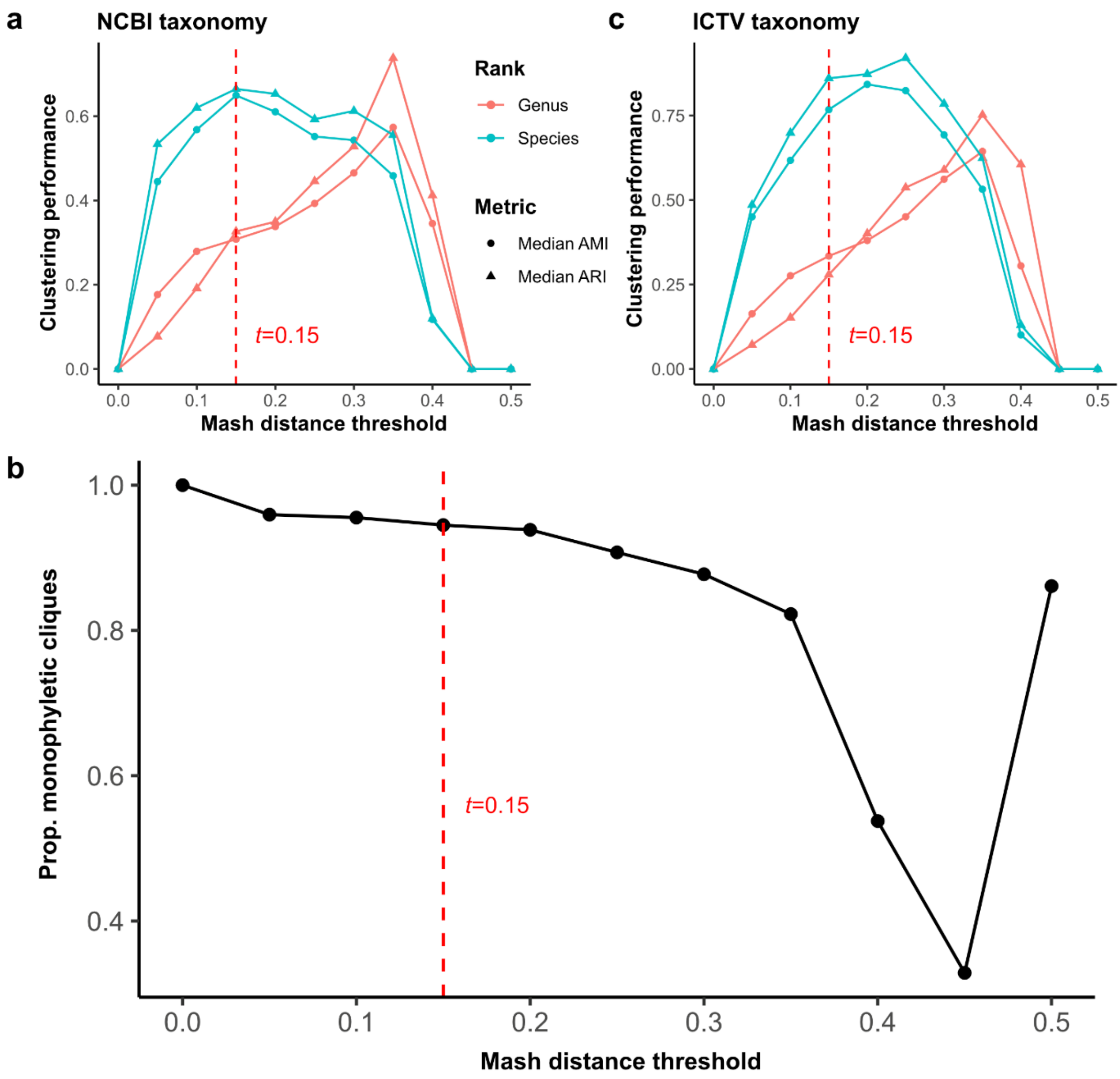

Supplementary Figure 2. Selection of Mash distance threshold. We constructed undirected, weighted graphs for each viral family with nodes and edges representing genomes and Mash distances, respectively. We then removed edges associated with values larger than the Mash distance threshold, *t*, before applying the community-detection algorithm, Infomap, to identify viral cliques. We then assessed the concordance of viral cliques identified against the (a) NCBI taxonomy or (c) ICTV taxonomy, using the median adjusted mutual information (AMI) or adjusted Rand index (ARI) across viral families. We also assessed (b) the overall proportion of monophyletic viral cliques across all viral families. The final distance threshold selected was *t*=0.15.

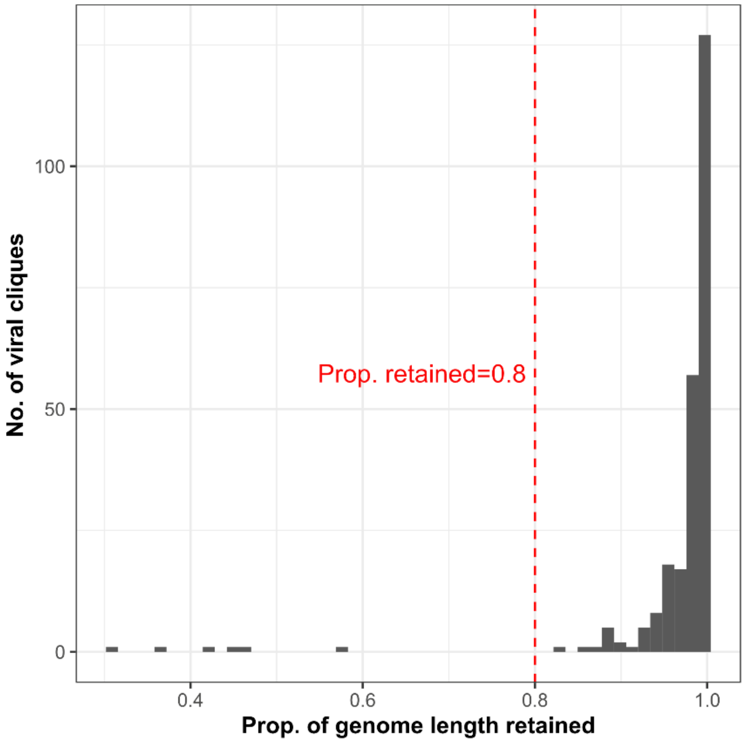

Supplementary Figure 3. Assessing the quality of clique-level genome alignments. For each viral clique, genomes were aligned and positions in these alignments were masked if more than 10% of the sequences corresponded to gaps or ambiguous nucleotides. The proportion of genome length retained was calculated as the number of unmasked sites divided by the median genome length of sequences in each clique-level alignment.

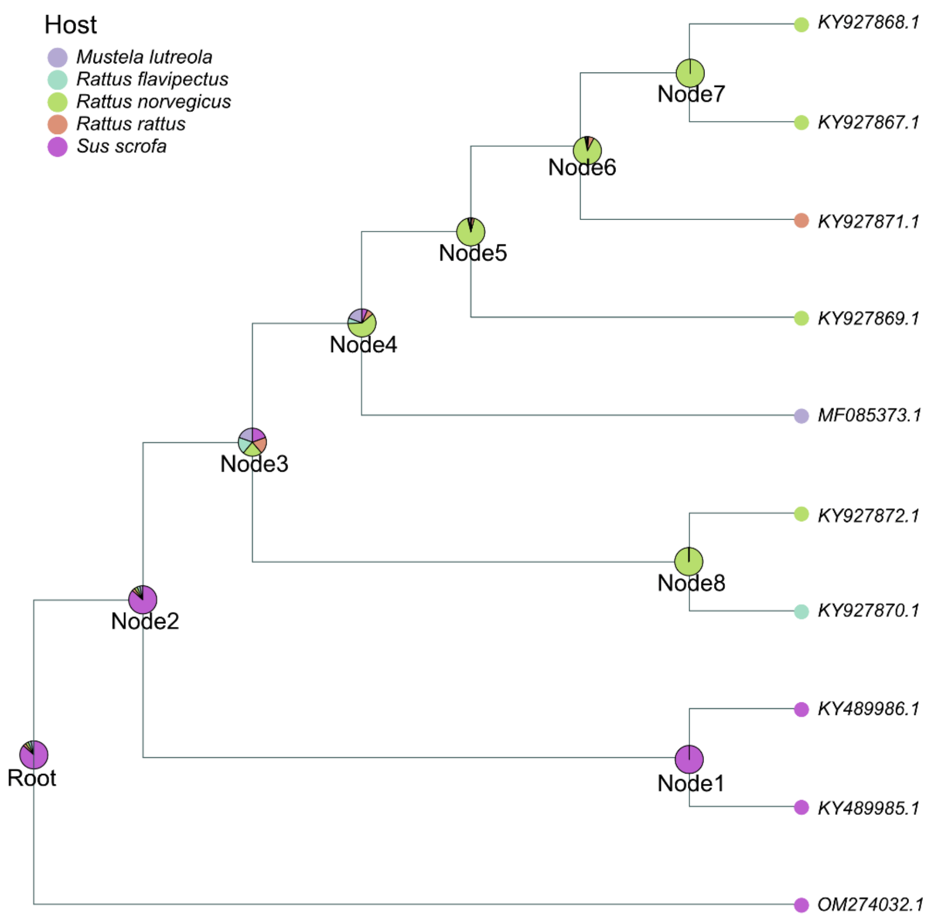

Supplementary Figure 4. Illustration of host jump inference. Maximum likelihood tree for the viral clique Parvoviridae_58, rooted at OM274032.1. Piecharts provide the relative likelihoods of all host states represented in this tree, as determined by maximum-likelihood ancestral state reconstruction.

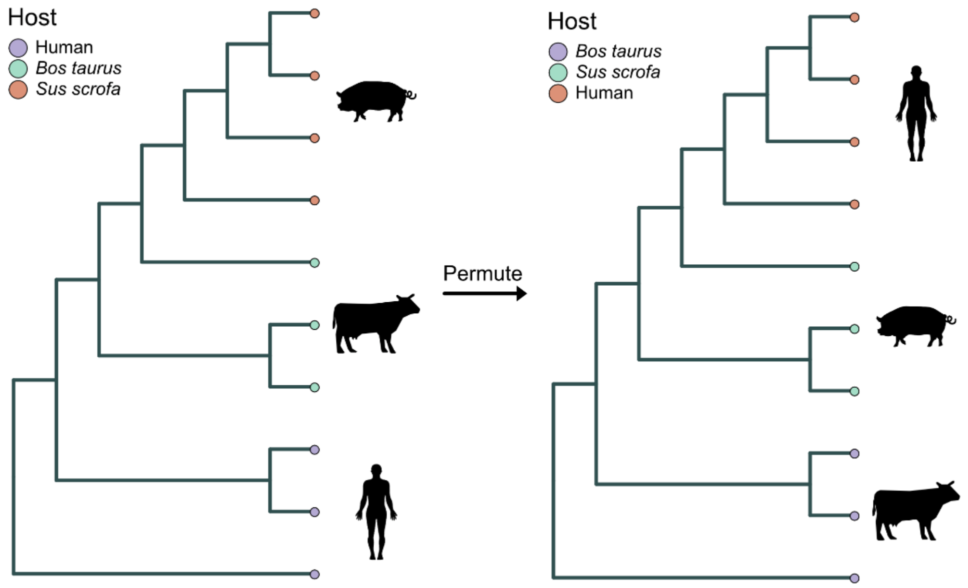

Supplementary Figure 5. Permutation test illustration. Illustration of our permutation test approach applied to a hypothetical tree of a viral clique associated to three distinct hosts. Host states are permuted while retaining the number of host jumps inferred.

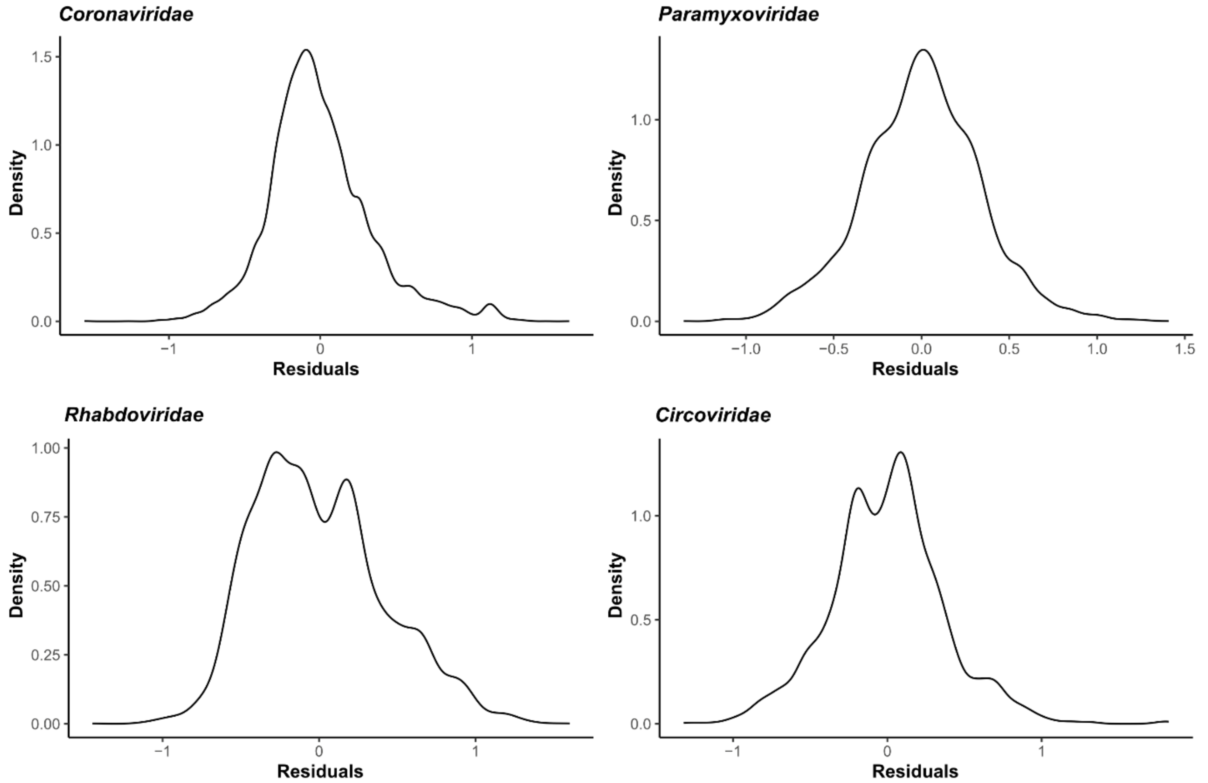

Supplementary Figure 6. Linear regression model residuals. Kernel density plots of residuals for the linear regression models implemented in Fig. 5.

References

1. Oreshkova, N. *et al.* SARS-CoV-2 infection in farmed minks, the Netherlands, April and May 2020. *Eurosurveillance* **25**, 2001005 (2020).
2. Munnink, B. B. O. *et al.* Transmission of SARS-CoV-2 on mink farms between humans and mink and back to humans. *Science* **371**, 172–177 (2021).
3. Tan, C. C. S. *et al.* Transmission of SARS-CoV-2 from humans to animals and potential host adaptation. *Nature Communications* **13**, 2988 (2022).
4. Chandler, J. C. *et al.* SARS-CoV-2 exposure in wild white-tailed deer (Odocoileus virginianus). *Proceedings of the National Academy of Sciences* **118**, e2114828118 (2021).
5. Kuchipudi, S. V. *et al.* Multiple spillovers from humans and onward transmission of SARS-CoV-2 in white-tailed deer. *Proceedings of the National Academy of Sciences* **119**, e2121644119 (2022).

6. Hale, V. L. *et al.* SARS-CoV-2 infection in free-ranging white-tailed deer. *Nature* **602**, 481–486 (2021).

7. Yen, H.-L. *et al.* Transmission of SARS-CoV-2 delta variant (AY. 127) from pet hamsters to humans, leading to onward human-to-human transmission: a case study. *The Lancet* **399**, 1070–1078 (2022).

8. Pickering, B. *et al.* Divergent SARS-CoV-2 variant emerges in white-tailed deer with deer-to-human transmission. *Nature Microbiology* **7**, 2011–2024 (2022).

9. Siegrist, A. A. *et al.* Probable Transmission of SARS-CoV-2 from African Lion to Zoo Employees, Indiana, USA, 2021. *Emerging Infectious Diseases* **29**, 1102 (2023).

10. Ran, Z. *et al.* Domestic pigs are susceptible to infection with influenza B viruses. *Journal of virology* **89**, 4818–4826 (2015).

11. He, W.-T. *et al.* Virome characterization of game animals in China reveals a spectrum of emerging pathogens. *Cell* **185**, 1117–1129 (2022).

12. Ye, R.-Z. *et al.* Natural infection of pangolins with human respiratory syncytial viruses. *Current Biology* **32**, R307–R308 (2022).

13. Kimura, H. *et al.* Interspecies transmission of influenza C virus between humans and pigs. *Virus research* **48**, 71–79 (1997).

14. Zhou, C. *et al.* The genome sequence of a novel simian adenovirus in a chimpanzee reveals a close relationship to human adenoviruses. *Archives of virology* **159**, 1765–1770 (2014).

15. Tan, B. *et al.* Isolation and characterization of adenoviruses infecting endangered golden snub-nosed monkeys (Rhinopithecus roxellana). *Virology Journal* **13**, 1–5 (2016).

16. Negrey, J. D. *et al.* Simultaneous outbreaks of respiratory disease in wild chimpanzees caused by distinct viruses of human origin. *Emerging Microbes & Infections* **8**, 139–149 (2019).

17. Mazet, J. A. *et al.* Human respiratory syncytial virus detected in mountain gorilla respiratory outbreaks. *EcoHealth* **17**, 449–460 (2020).

18. Medkour, H. *et al.* Adenovirus infections in African humans and wild non-human primates: great diversity and cross-species transmission. *Viruses* **12**, 657 (2020).

19. Jiang, B., McClure, H. M., Fankhauser, R. L., Monroe, S. S. & Glass, R. I. Prevalence of rotavirus and norovirus antibodies in non-human primates. *Journal of Medical Primatology* **33**, 30–33 (2004).

20. Bwogi, J. *et al.* Whole genome analysis of selected human and animal rotaviruses identified in Uganda from 2012 to 2014 reveals complex genome reassortment events between human, bovine, caprine and porcine strains. *PloS one* **12**, e0178855 (2017).

21. Negrey, J. D. *et al.* Viruses associated with ill health in wild chimpanzees. *American Journal of Primatology* **84**, e23358 (2022).

22. Sam, I.-C. *et al.* Chikungunya virus in macaques, Malaysia. *Emerging Infectious Diseases* **21**, 1683 (2015).
