# Supplementary material for: The evolutionary drivers and correlates of viral host jumps
Source: Nat Ecol Evol. 2024 Mar 25;8(5):960–71. doi: 10.1038/s41559-024-02353-4 (PMC11090819; doi:10.1038/s41559-024-02353-4)
Supplement: Supplementary file 2 — Reporting Summary [file 41559_2024_2353_MOESM2_ESM.pdf]

nature portfolio

Corresponding author(s): Cedric C.S. Tan

Last updated by author(s): Jan 19, 2024

Reporting Summary

Nature Portfolio wishes to improve the reproducibility of the work that we publish. This form provides structure for consistency and transparency in reporting. For further information on Nature Portfolio policies, see our Editorial Policies and the Editorial Policy Checklist.

Statistics

For all statistical analyses, confirm that the following items are present in the figure legend, table legend, main text, or Methods section.

n/a	Confirmed	
☐	☒	The exact sample size (*n*) for each experimental group/condition, given as a discrete number and unit of measurement
☒	☐	A statement on whether measurements were taken from distinct samples or whether the same sample was measured repeatedly
☐	☒	The statistical test(s) used AND whether they are one- or two-sided<br>*Only common tests should be described solely by name; describe more complex techniques in the Methods section.*
☐	☒	A description of all covariates tested
☐	☒	A description of any assumptions or corrections, such as tests of normality and adjustment for multiple comparisons
☐	☒	A full description of the statistical parameters including central tendency (e.g. means) or other basic estimates (e.g. regression coefficient) AND variation (e.g. standard deviation) or associated estimates of uncertainty (e.g. confidence intervals)
☐	☒	For null hypothesis testing, the test statistic (e.g. *F*, *t*, *r*) with confidence intervals, effect sizes, degrees of freedom and *P* value noted<br>*Give P values as exact values whenever suitable.*
☒	☐	For Bayesian analysis, information on the choice of priors and Markov chain Monte Carlo settings
☒	☐	For hierarchical and complex designs, identification of the appropriate level for tests and full reporting of outcomes
☐	☒	Estimates of effect sizes (e.g. Cohen's *d*, Pearson's *r*), indicating how they were calculated

Our web collection on statistics for biologists contains articles on many of the points above.

Software and code

Policy information about availability of computer code

Data collection	taxizedb v0.3.1<br>ncbi-acc-download v0.2.8
Data analysis	R v4.3.1<br>CheckV v1.0.1<br>Mash v1.1<br>Ape v5.7.1<br>Aricode v1.0.2<br>MAFFT v7.490<br>IQ-Tree v2.1.4-beta<br>Castor v1.7.10<br>MSA2dist v1.4.0<br>ggtree v3.8.2<br>ggplot v3.4.2

For manuscripts utilizing custom algorithms or software that are central to the research but not yet described in published literature, software must be made available to editors and reviewers. We strongly encourage code deposition in a community repository (e.g. GitHub). See the Nature Portfolio guidelines for submitting code & software for further information.

Data

Policy information about availability of data

All manuscripts must include a data availability statement. This statement should provide the following information, where applicable:

- Accession codes, unique identifiers, or web links for publicly available datasets
- A description of any restrictions on data availability
- For clinical datasets or third party data, please ensure that the statement adheres to our policy

All custom code used to perform the analyses reported here are hosted on GitHub (https://github.com/cednotsed/vertebrate_host_jumps). The full list of accessions considered in this study are provided in Supplementary Table 1.

Research involving human participants, their data, or biological material

Policy information about studies with human participants or human data. See also policy information about sex, gender (identity/presentation), and sexual orientation and race, ethnicity and racism.

Reporting on sex and gender	N.A.
Reporting on race, ethnicity, or other socially relevant groupings	N.A.
Population characteristics	N.A.
Recruitment	N.A.
Ethics oversight	N.A.

Note that full information on the approval of the study protocol must also be provided in the manuscript.

Field-specific reporting

Please select the one below that is the best fit for your research. If you are not sure, read the appropriate sections before making your selection.

☒ Life sciences ☐ Behavioural & social sciences ☐ Ecological, evolutionary & environmental sciences

For a reference copy of the document with all sections, see nature.com/documents/nr-reporting-summary-flat.pdf

Life sciences study design

All studies must disclose on these points even when the disclosure is negative.

Sample size	No sample size calculation was performed. We used almost all publicly available genome sequences relevant to this study. Approximately 56k genomes were used for our analyses, and at least 500 genomes per group (i.e., viral family) were used, so sample sizes are sufficient.
Data exclusions	To generate a candidate list of viral sequences for further genomic analysis, the metadata was filtered to include 53 viral families known to infect vertebrate hosts based on information provided in the 2022 release of the ICTV taxonomy (https://ictv.global/taxonomy), and with reference to that provided by ViralZone (https://viralzone.expasy.org/). We then retained only sequences from viral families comprising at least 100 sequences of greater than 1000nt in length. For non-segmented genomes, we retained all non-human-associated sequences, and subsampled the human-associated sequences as follows: we selected a random subsample of 1000 SARS-CoV-2 genomes of greater than 28000nt from distinct countries, isolation sources, and with distinct collection dates. For other human-associated sequences, we retained viruses with distinct species, country, isolation source and collection date information. . We then downloaded the final candidate list of viral sequences (n=88,161) using the ncbi-acc-download v0.2.8 (https://github.com/kblin/ncbi-acc-download). Further quality control of the genomes downloaded was performed using CheckV v1.0.146, retaining sequences with more than 95% completeness (for non-segmented viruses) and less than 5% contamination (for all sequences). This resulted in a final genomic dataset comprising 53,631 observations (Supplementary Table 2).<br><br>For clique-level alignments, we masked regions of the alignments that were poorly aligned or prone to sequencing-error by replacing alignment sites that had more than 10% of gaps or ambiguous nucleotides with N's. Clique-level genome alignments that had more than 20% of the median genome length masked were considered to be poorly aligned and removed from further analysis (n=6; Extended Data Fig. 5)
Replication	No experimental findings were reported so this section is not applicable.
Randomization	Genomic datasets used for our study are retrospective and downloaded from public sequence databases so randomisation is not applicable to our study.
Blinding	Genomic datasets used for our study are retrospective and downloaded from public sequence databases so blinding is not applicable to our study.

Reporting for specific materials, systems and methods

We require information from authors about some types of materials, experimental systems and methods used in many studies. Here, indicate whether each material, system or method listed is relevant to your study. If you are not sure if a list item applies to your research, read the appropriate section before selecting a response.

Materials & experimental systems

n/a	Involved in the study
☒	☐ Antibodies
☒	☐ Eukaryotic cell lines
☒	☐ Palaeontology and archaeology
☒	☐ Animals and other organisms
☒	☐ Clinical data
☒	☐ Dual use research of concern
☒	☐ Plants

Methods

n/a	Involved in the study
☒	☐ ChIP-seq
☒	☐ Flow cytometry
☒	☐ MRI-based neuroimaging
